# Grapes and Their Derivatives in Functional Foods

**DOI:** 10.3390/foods10030672

**Published:** 2021-03-22

**Authors:** Panagiotis Kandylis

**Affiliations:** Laboratory of Oenology and Alcoholic Beverages, Department of Food Science and Technology, School of Agriculture, Aristotle University of Thessaloniki, P.O. Box 235, 54124 Thessaloniki, Greece; pkandylis@agro.auth.gr; Tel.: +30-2310-991678

Consumer interest in the consumption of health promoting foods is growing worldwide due to the realization of the link between diet and human health. This has led to the development of a new category of foods, the so-called functional foods. Since not all foods contain ingredients with functional properties or at the desired concentrations, their fortification with such functional ingredients is a necessity. Grapes (or other fruits with phenolic compounds) and their derivatives have the potential to be used in order to increase the functional properties of several foods or to design novel functional foods [1,2].

Grapes have a long history and have been part of the human diet for more than 6000 years. Through centuries they have been used in several forms, such as directly as food (table grapes), for the production of juice and wines (wine grapes), or as dried grapes for longer shelf life (raisin grapes). The processing of grapes, especially for wine production, results in a large amount of different wastes such as grape pomace, grape seeds, wine lees, etc. However, these wastes still contain various ingredients of grapes that may provide health benefits beyond nutrition, especially their phenolic compounds. Furthermore, not only does wine making result in a large amount of waste, but also the practices (vine pruning) in the vineyard. Vine pruning residues remain under-exploited and are often burned in the fields, contributing to green-house gas emissions. The importance of these by-products is well known, and methods for their sustainable exploitation in wine making have been proposed [2].

This *Special Issue* is devoted to the presentation of several recent applications of grapes (or other fruits with phenolic compounds) and their derivatives in the development of functional foods.

Raw material is the most important factor for the quality of the final product, but also for the quality of a possible by-product. For grapes, it is very important to evaluate the ideal conditions of harvesting that result in a high-quality product. Within this context, Mucalo et al. [3] evaluated the effect of an extended harvest on the flavonoid composition and chromatic characteristics of Plavac Mali grape berries. The extended harvest changed the flavonoid composition in favor of anthocyanins and flavonols. Finding the optimum time for grape harvest is a technological challenge as conventional indicators of ripeness (sugar, total acidity, and pH) and the polyphenolic indicators (anthocyanins, flavonols and monomer, and dimer proanthocyanidin units) split up significantly with different optimal harvest dates.

One method to exploit valuable by-products such as grape pomace is their inclusion in the diet of animals in order to increase the nutritional value of their meat. As an example, the inclusion of grape pomace in poultry diet resulted in improved nutritional value due to the increase in the polyunsaturated fatty acids (PUFA): saturated fatty acids (SFA) ratio and extended shelf-life due to improvement in the oxidative stability [4]. In a similar manner, dietary grape pomace has great potential to be incorporated into the diet of dairy cows, and thus positively affects the nutritional properties of their milk and other dairy products. More specifically, milk and dairy products are characterized by implemented nutritional properties and improved oxidative stability, with several health benefits for consumers due to the presence of compounds credited with high biological value. Furthermore, an improvement of their organoleptic properties is reported, which may increase consumer acceptability [5].

Another approach is the incorporation of grape derivatives into foods in order to increase their functional characteristics. Dairy products [6,7] and meat products [8] are the ideal carriers of these compounds. Grape derivatives such as grape juice may be incorporated into the production of yogurts resulting in increased total phenolic content and antioxidant capacity [6]. In the same manner, grape skin extract, together with inulin and fructo-oligosaccharides, proved suitable ingredients for the development of yogurts with antioxidant and antidiabetic properties, but also gained the acceptance of consumers [7]. Furthermore, grape seed extract may be used as natural antioxidant in the production of dry-fermented pork necks inoculated with probiotic bacteria [8]. 

Grape products and by-products have great health promoting properties. Even vine pruning residue is a possible carrier of health promoting compounds. An ohmic extract of vine pruning characterized by its high polyphenol content proved capable of possessing anti-colorectal cancer properties, including sensitization to a chemotherapeutical drug. Thus, its use in functional foods or nutraceuticals should be exploited, particularly in the diet of patients following specific anti-colorectal cancer strategies [9]. Furthermore, grape skin extract with a high content of phenolic compounds (flavonoids, phenolic acids, and phenolic alcohols) may have the potential to reduce the risk of diabetes. Indeed, the bioaccessible compounds of grape skin extract proved capable of modulating the key biochemical events involved in the pathogenesis of diabetes, such as oxidative stress, inflammation, and glucose absorption [10].

However, it is not only grapes that have the potential to be used in functional products due to their numerous health properties as other fruit-based products such as pomegranates also have a similar increased phenolic content and high antioxidant capacity [1].

The interest of consumers for foods with high nutritional value and possible health promoting properties is continuously increasing, and therefore further expansion is also expected in the sector of the exploitation of grapes and their derivatives. Therefore, more research is needed in order to have a better understanding of the health promoting properties of such by-products and the mechanism of action. Furthermore, their incorporation in foods may result in changes in their organoleptic characteristics, which should be extensively studied.
